# Detection of tomato water stress based on terahertz spectroscopy

**DOI:** 10.3389/fpls.2023.1095434

**Published:** 2023-01-30

**Authors:** Yixue Zhang, Xinzhong Wang, Yafei Wang, Lian Hu, Pei Wang

**Affiliations:** ^1^ Basic Engineering Training Center, Jiangsu University, Zhenjiang, China; ^2^ College of Agricultural Engineering, Jiangsu University, Zhenjiang, China; ^3^ Key Laboratory of Key Technology on Agricultural Machine and Equipment, Ministry of Education, South China Agricultural University, Guangzhou, China

**Keywords:** terahertz spectroscopy, spectroscopy, terahertz, tomato, water stress

## Abstract

China’s tomato cultivation area is nearly 15 thousand km^2^, and its annual tomato output is about 55 million tons, accounting for 7% of its total vegetable production. Because of the high drought sensitivity of tomatoes, water stress inhibits their nutrient uptake, leading to a decrease in tomato quality and yield. Therefore, the rapid, accurate and non-destructive detection of water status is important for scientifically and effectively managing tomato water and fertilizer, improving the efficiency of water resource utilization, and safeguarding tomato yield and quality. Because of the extreme sensitivity of terahertz spectroscopy to water, we proposed a tomato leaf moisture detection method based on terahertz spectroscopy and made a preliminary exploration of the relationship between tomato water stress and terahertz spectral data. Tomato plants were grown at four levels of water stress. Fresh tomato leaves were sampled at fruit set, moisture content was calculated, and spectral data were collected through a terahertz time-domain spectroscope. The raw spectral data were smoothed using the Savitzky–Golay algorithm to reduce interference and noise. Then the data were divided by the Kennard–Stone algorithm and the sample set was partitioned based on the joint X-Y distance (SPXY) algorithm into a calibration set and a prediction set at a ratio of 3:1. SPXY was found to be the better approach for sample division. On this basis, the stability competitive adaptive re-weighted sampling algorithm was used to extract the feature frequency bands of moisture content, and a multiple linear regression model of leaf moisture content was established under the single dimensions of power, absorbance and transmittance. The absorbance model was the best, with a prediction set correlation coefficient of 0.9145 and a root mean square error of 0.1199. To further improve the modeling accuracy, we used a support vector machine (SVM) to establish a tomato moisture fusion prediction model based on the fusion of three-dimensional terahertz feature frequency bands. As water stress intensified, the power and absorbance spectral values both declined, and both were significantly and negatively correlated with leaf moisture content. The transmittance spectral value increased gradually with the intensification of water stress, showing a significant positive correlation. The SVM-based three-dimensional fusion prediction model showed a prediction set correlation coefficient of 0.9792 and a root mean square error of 0.0531, indicating that it outperformed the three single-dimensional models. Hence, terahertz spectroscopy can be applied to the detection of tomato leaf moisture content and provides a reference for tomato moisture detection.

## 1 Introduction

Water resources are the key to agricultural development, and China’s per capita water resource appropriation is extremely low: only one quarter of the global average. As a large agricultural country, agricultural water consumption accounts for 65% of total water consumption in China, but its effective utilization rate is only 45%, far lower than the levels of 70%–80% in advanced water-saving countries (Zhang et al., 2020). At the same time, the production approach of “big water and big fertilizer” also leads to a reduction in crop quality. Therefore, vigorously improving the efficiency of agricultural water use is an important strategic measure to ensure water security and improve crop quality in China.

As one of the main facility crops, tomatoes are planted throughout China. In 2017, China’s facility tomato planting area was nearly 15 thousand km^2^ (Wang et al., 2021), and its annual production accounted for nearly 1/3 of the global tomato output. Tomatoes require a long growth period and a large amount of water, and because of their extreme sensitivity to water, they have specific requirements for water at different growth stages. Insufficient water inhibits the absorption of nutrients by crops and slows the growth of leaf area, plant height and stem thickness, resulting in reduced crop quality and yield (Zhang et al., 2023). Therefore, the rapid, accurate and non-destructive detection of tomato water status is very important for scientifically and effectively managing tomato water and fertilizer use, improving water resource utilization efficiency, and ensuring tomato yield and quality in China.

Nondestructive detection of crop water stress has been studied extensively, and the most important methods include spectroscopy, the infrared canopy temperature method, and imaging. Fitzgerald et al. (2006) tested the ability of several multispectral indexes to estimate the nutrient status of the wheat canopy grown under different degrees of water stress and derived the canopy reflectance index that was closely related to these factors. Although moisture status was diagnosed, no quantitative moisture detection method was established. Huang et al. (2009) analyzed the factors that affected the accuracy of a near-infrared (NIR) spectral straw moisture model, introducing the LOCAL algorithm for nondestructive detection of straw moisture and establishing an NIR straw moisture prediction model. Nevertheless, the influence of environmental factors such as changes in natural light intensity was ignored, suggesting that the detection accuracy required further improvement. Zhang et al. (2018) used drones equipped with thermal infrared sensors for temperature detection in cotton fields and found that cotton canopy temperature characteristics were correlated with cotton moisture. However, the use of drones is greatly influenced by environmental uncertainties such as ground evaporation, environmental heat exchange, and airflow, and the stability and accuracy of the model are low. Tian et al. (2016) used NIR hyperspectral imaging to extract images of maize grain embryo structure and established a maize grain moisture content prediction model. Unfortunately, the sample processing and analysis process was relatively complicated, which is not conducive to rapid moisture detection. The studies above show that although NIR hyperspectral and thermal infrared data show good correlation with water stress, they are greatly influenced by field energy exchange, solar radiation, and other environmental changes because of the use of thermal radiation detection. These methods often cannot comprehensively describe the physical characteristics or internal tissue physiological and biochemical characteristics of leaves under water stress, which undoubtedly impacts the accuracy of the measurements.

Terahertz spectroscopy has been described as one of the ten technologies that will influence the future of mankind in the 21st century (Zhang et al., 2022). Terahertz waves are electromagnetic waves with the frequency between 0.1 and 10 THz and a wavelength range of 30 μm to 3 mm; they lie between microwave and infrared radiation on the electromagnetic spectrum (Luo et al., 2019). Terahertz radiation is penetrating, fingerprinting, and coherent, and it has the advantage of multi-dimensional fusion detection. Traditional spectroscopic and imaging methods typically obtain information about only the reflection characteristics of the detected object and its distribution (image) in different characteristic spectral bands. By comparison, terahertz time-domain spectroscopy is rich in information about the substance (Wang et al., 2017). Under terahertz radiation, polar molecules such as water undergo hydrogen bond breaking and formation on picosecond timescales, leading to intense absorption of terahertz waves (Yada et al., 2008; Yang et al., 2018). The terahertz technique can be used to detect the water status of crops based on this phenomenon.

Terahertz spectroscopy has been applied to nondestructive moisture detection. Because the absorption of terahertz spectra by proteins, amino acids, and other substances in biscuits is much lower than that of water, Liu et al. (Liu and Han, 2014) modeled the frequency domain, refractive index, and absorption coefficient of terahertz spectra by principal component analysis (PCA) and partial least squares separately, and the absorption coefficient was better than the other spectra. Breitenstein et al. (2011) examined water stress in coffee plants and demonstrated the great potential and reliability of terahertz spectroscopy for monitoring leaf moisture content in the field. Gente et al. (Gente et al., 2013; Gente et al., 2015) established a moisture prediction model for barley leaves by combining the transmission and absorption coefficients in the terahertz spectra, and its results were consistent with the true leaf moisture content. Long et al. (2017) scanned green herb leaves *in vitro* point-by-point at intervals using a terahertz spectrometer and reconstructed images to observe the differences in moisture content. They established a regression prediction model based on water content and the time-domain and frequency-domain mean values of the images, thereby demonstrating the applicability of terahertz technology to leaf moisture detection. Zhao et al. (2018) studied soybean canopy moisture content using terahertz spectroscopy by simulating drought stress. They used partial least squares and multiple linear regression to establish a correlation model of the time-domain spectra, absorption coefficient, and refractive index with leaf moisture content, providing a solution for rapid monitoring of soybean canopy water content. The studies above show the potential of terahertz spectroscopy for moisture content detection.

Nondestructive detection based on terahertz time-domain spectroscopy has the advantages of speed, convenience, and minimal disturbance compared with other detection methods. Existing studies reveal a significant correlation between terahertz spectroscopy data and crop moisture content, and the use of terahertz spectroscopy holds promise for water stress detection.

Leaf moisture content is an important indicator for diagnosing tomato water stress. In this study, we cultivated tomatoes at different levels of water stress and used terahertz spectroscopy to acquire time-domain terahertz spectra of tomato leaves, including the power spectrum, absorbance, and transmittance. A high-precision tomato leaf moisture content prediction model was established. This study provides a basis for scientific and appropriate precision management of water and fertilizer during tomato cultivation.

## 2 Materials and methods

### 2.1 Sample cultivation

The quality of the test sample cultivation has a direct impact on the test results. Therefore, in the process of sample cultivation, the influence of environmental factors should be minimized and the accuracy of the sample data should be improved. The experiment was carried out in a Venlo-type greenhouse (32.2°N, 119.5°E) at the Key Laboratory of Modern Agricultural Equipment and Technology, Ministry of Education, Jiangsu University. The environmental temperature of the greenhouse was maintained at 10.7-29.4°C, and the relative humidity was 37.3%-87.9%. The test samples were 906 red tomatoes (Shanghai Changchong Tomato Seed Industry Co., Ltd.). Tomato seeds with large, plump grains and similar shapes were selected, and the selected seeds were placed in lightly salted water to screen out diseased seeds and sclerotia. The experimental samples were cultivated in a soilless pot with nutrient solution based on perlite.

In order to study the condition of tomatoes under different water stress and accurately control the water under the condition of ensuring the balance of nutrient elements, four water stress gradients were set according to 20%, 40%, 60% and 80% of the standard irrigation (600 mL, a mixture of water and nutrient solution) amount from the 5th day of planting, and 10 pots of tomato samples were cultivated in each gradient. During the experiment, the cultured tomato plants were watered with the Japanese Yamazaki nutrient solution formula.

### 2.2 Instruments and equipment

**Figure 1:**
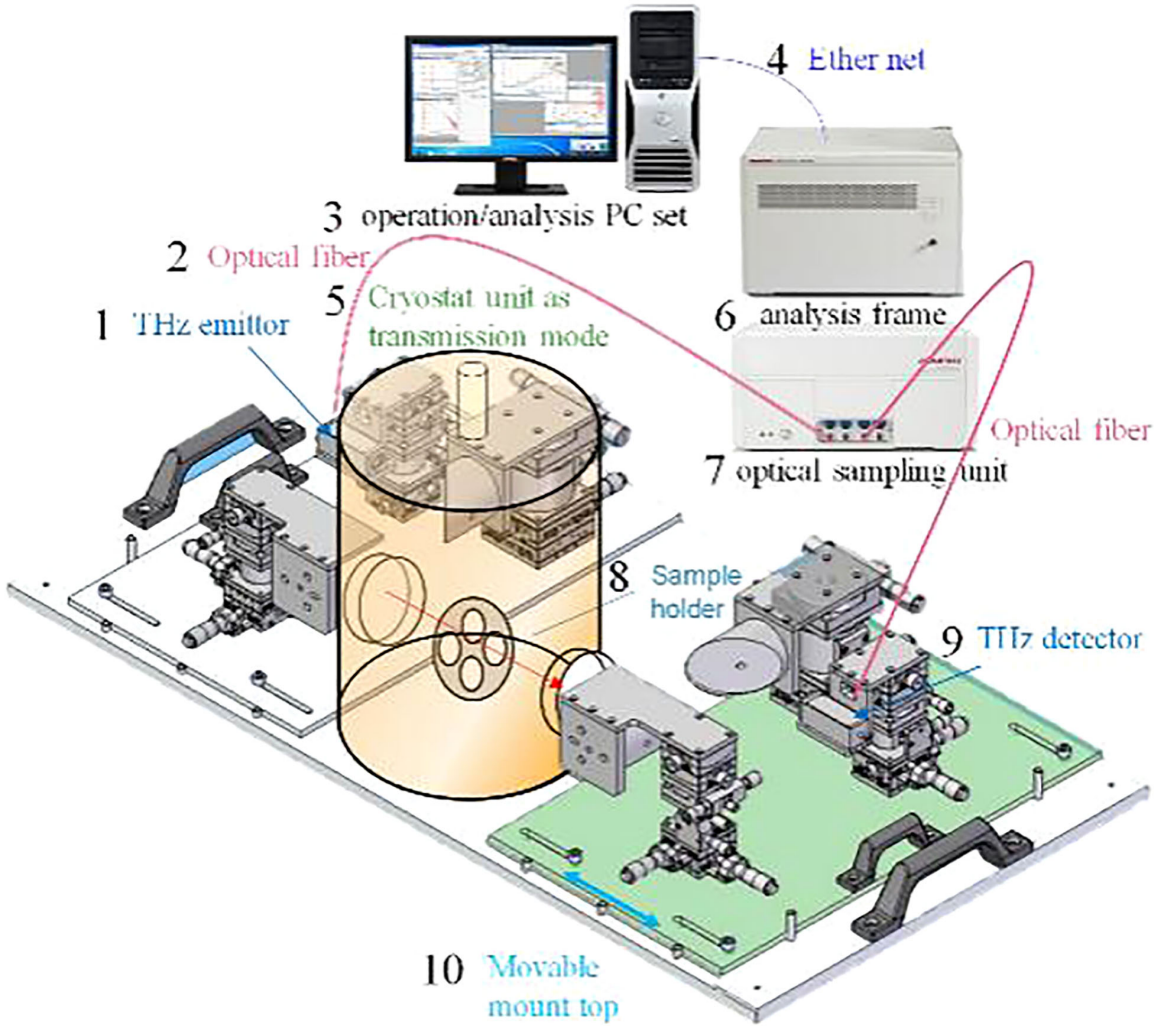
The structure and working principle of the Advantest-TS7400 THz-TDS measurement system.

In this study, the TS7400 THz time-domain spectral measurement system produced by Edevan Company of Japan is used to collect the terahertz information of samples. This system is specially customized for agricultural biological information detection. It has an ART attenuated total reflection module that can detect biological tissues and living samples with high water content. **Figure 1** shows the structure and working principle of the TS7400 THz time-domain spectral measurement system. When the measurement system is working, the laser light source emits laser pulses. Under the action of the beam splitter, it is divided into two mutually perpendicular lasers. One is a strong pump light, and the other is a weak probe light. The pump light passes through the terahertz transmitter and reflector, passes through the measured sample, and then, collinear with the multiple reflected probe light, passes through the probe crystal and is transmitted to the terahertz detector. The detector transmits the difference between the two laser beams to the A/D module, The time-domain terahertz spectrum and its distribution information of the samples are obtained by comparing their differences through data processing.

1. Operating/analyzing computers; 2. Ethernet; 3. Optical fiber; 4. Analysis unit; 5. Measurement unit; 6. THz transmitter; 7. THz detector; 8. Sample stage; 9. Cryostat transfer module; 10. Removable stand.

Compared with the traditional terahertz device, the device not only has higher accuracy, but also can detect samples with a scale of up to 3 cm² Expand to 225 cm², It can better meet the measurement needs of crop samples. The measurement frequency range of TS7400 THz time-domain spectrum measurement system is 0-4 Thz, the resolution is less than or equal to 5 Ghz, the sampling interval is 0.0038 Thz, and the maximum sample area is 150 * 150 mm^2^.

The quality of tomato leaf samples was weighed with a high-precision analytical balance with an accuracy of 0.1 mg.

### 2.3 Data collection

Tomato leaf samples were collected 65 days after water stress treatment: healthy leaves were cut from the pinnate leaves of tomato plants that were most representative of the growth state. The leaves were immediately placed in fresh sealed bags and stored in a portable refrigerated incubator to prevent evaporation. Twenty leaf samples were selected from each water stress treatment for a total of 80 samples across all treatments.

After collection, the fresh leaves were weighed in a laboratory environment and then placed in a THz time-domain spectroscopy system to scan the spectra. To eliminate the influence of water vapor in the air on the THz spectra, we turned on the dehumidifier in advance to regulate the relative humidity in the test chamber to less than 5%. Each sample was scanned at 10 sampling points, and the data were averaged. After scanning, the leaves were dried in a 70°C incubator for more than 24 h. When sample quality no longer changed, the dry leaves were weighed. The moisture content of the tomato leaves (*w*, %) was calculated as:


(1) $$w=\frac{m_{1}-m_{2}}{m_{2}}x100$$

where *m*_1_ and *m*_2_ are the fresh and dry weight (g) of the sample, respectively.

## 3 Results and analysis

### 3.1 Terahertz spectrum analysis

#### 3.1.1 Power

**Figure 2:**
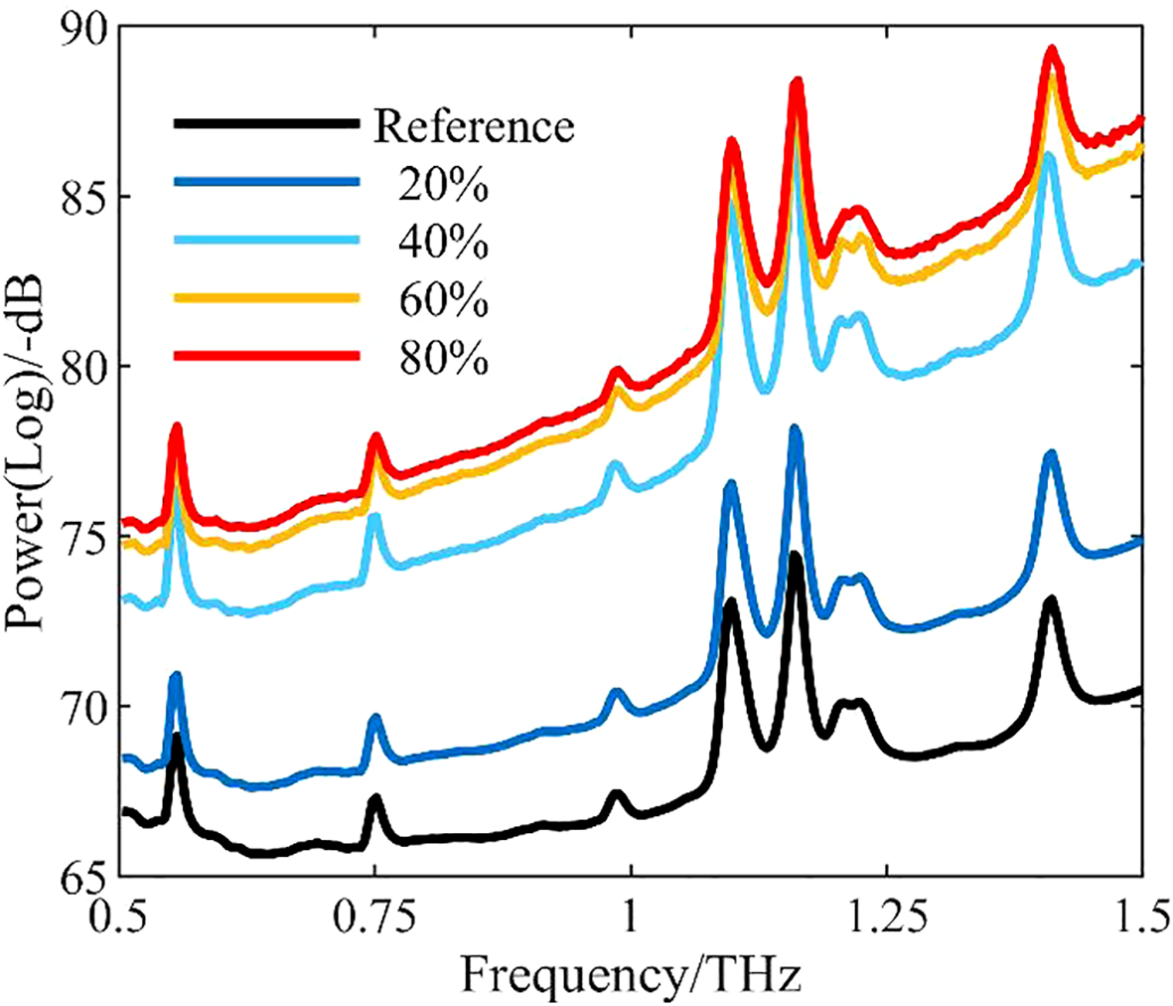
Curves of mean power measured for different water levels. Reference is the background data collected when no leaf samples were present.

The power spectrum density function, or power, is defined as the signal power per unit frequency band. It represents the variation of signal power with frequency, i.e., the distribution of signal power in the frequency domain (Du et al., 2022). The power spectrum can be used to find the relationship between signal power and moisture content. **Figure 2** shows the curves of mean power for different water levels in the frequency range of 0.5–1.5 THz.

As the leaf water level decreased, the mean curve of the power spectrum also decreased. The power spectra from leaves of different water contents clearly differed, and all were higher than the background data.

#### 3.1.2 Absorbance

**Figure 3:**
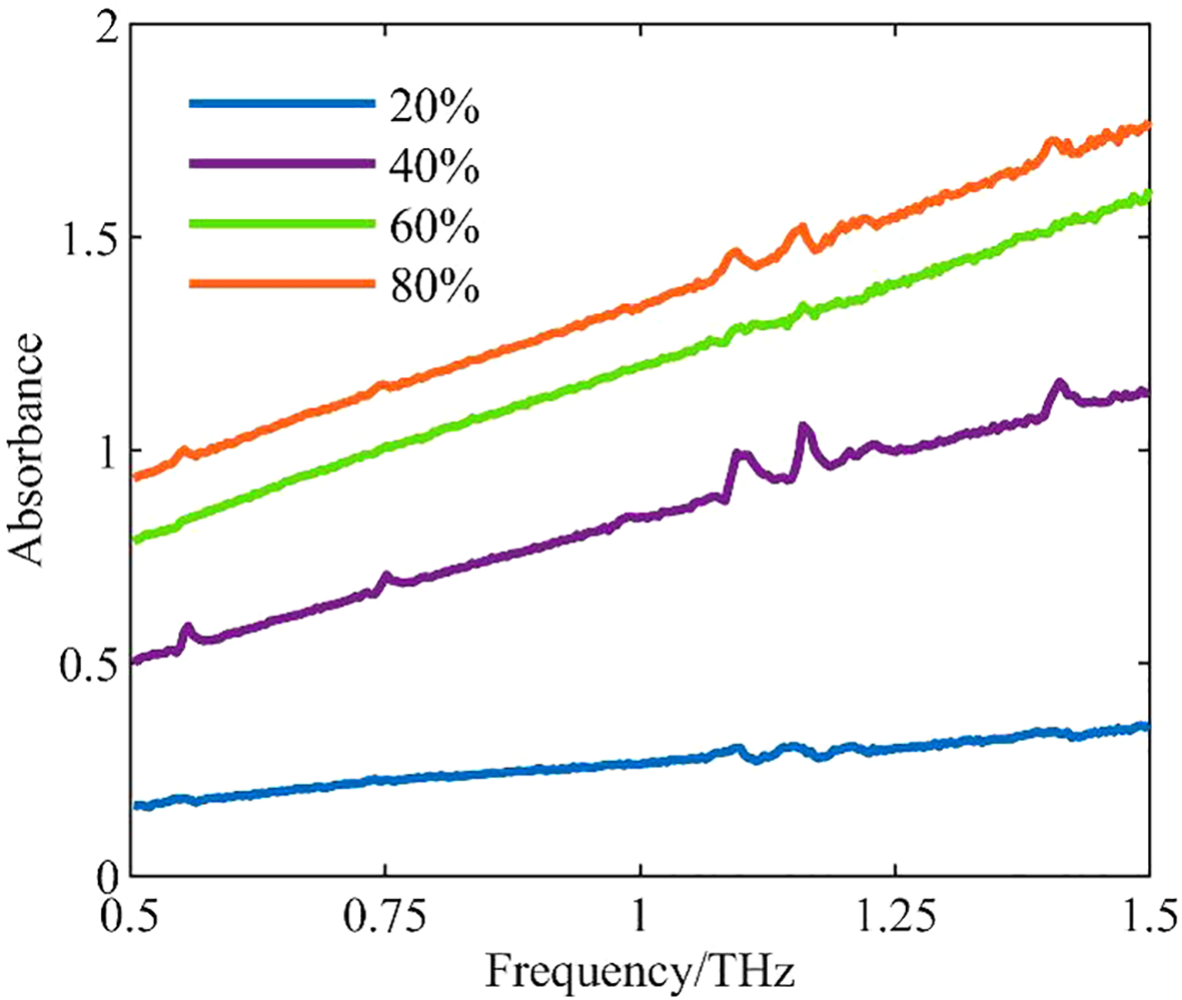
Curves of mean absorbance for different leaf water levels.

Absorbance reflects the degree of light absorption by a substance, and differences in moisture content among leaves cause differences in absorbance. **Figure 3** shows the curves of mean absorbance for different leaf water levels in the frequency range of 0.5–1.5 THz.

The water level of a sample was positively correlated with the absorbance and frequency of its terahertz spectrum. The higher the moisture content of the tomato leaf, the greater the absorbance, and absorbance clearly increased with increasing frequency. Clear differences were observed among different water levels, and there was a significant correlation.

#### 3.1.3 Transmittance

**Figure 4:**
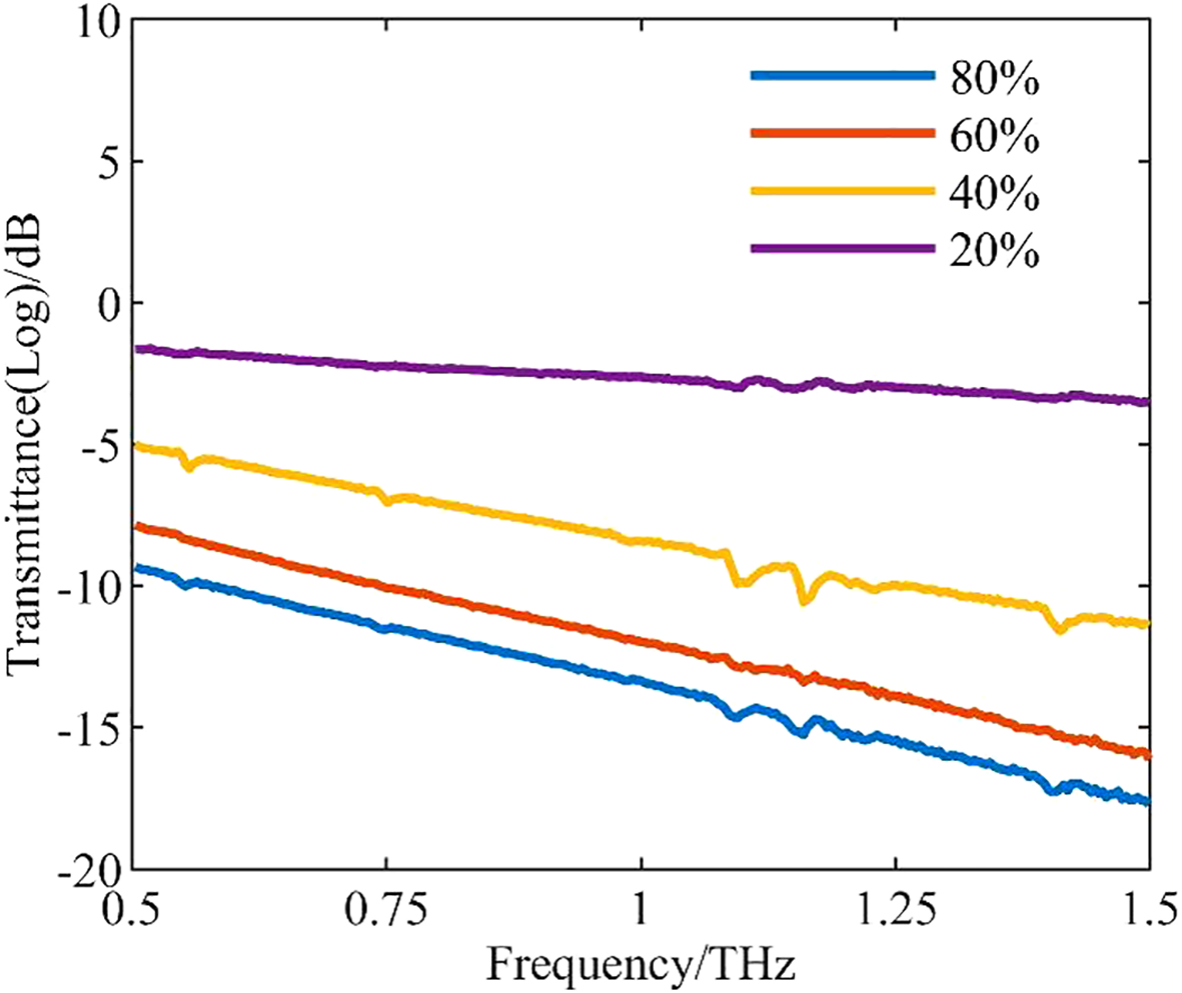
Curves of mean transmittance for leaves of different water levels.

Transmittance indicates the light transmission property of a sample. Because terahertz waves are sensitive to water, samples with different water levels differ significantly in transmittance. **Figure 4** shows the curves of mean transmittance for different water levels in the frequency range of 0.5–1.5 THz.

In this frequency range, transmittance decreases with increasing frequency. The lower the leaf water level, the higher the transmittance, and vice versa. The main reason for this phenomenon is that multiple interactions between polar molecules (e.g., water) occur during irradiation by terahertz waves, resulting in strong absorption of the terahertz waves (Yang et al., 2014).

### 3.2 Spectral modeling

#### 3.2.1 Data preprocessing

Terahertz time-domain spectral data will carry some noise during the acquisition process, and the raw data contain much redundant and invalid information and more interference noise. Hence, the data must be preprocessed to effectively reduce interference and improve the modeling efficiency and accuracy.

Here, the Savitzky–Golay (SG) smoothing algorithm was used to pre-process the data. The choice of window width and polynomial order are important when applying this algorithm; if the choice is inappropriate, the effect of filtering and the accuracy of the data will be affected.

**Figure 5:**
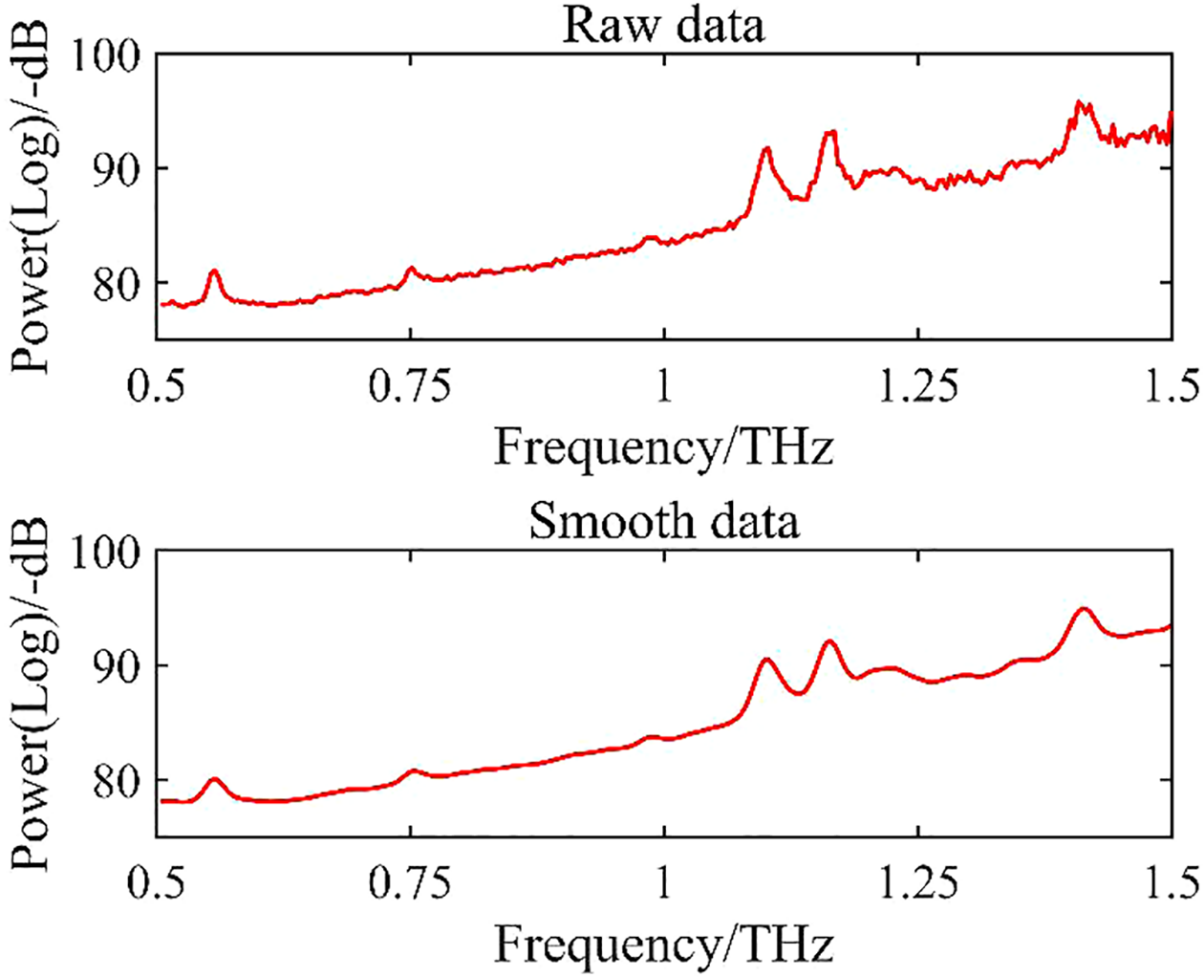
Comparison of data before and after smoothing.

Using power spectra as an example, **Table 1** shows the Rc and RMSE of the power spectra for the regression model of measured moisture content in the same frequency band after SG smoothing using windows of different widths. **Figure 5** shows a comparison of THz power data before and after smoothing.

**Table 1:** Preprocessing results with different window widths.

Points	5	7	9	11
*R*c	0.8804	0.8605	0.8452	0.8097
RMSE	0.1432	0.1692	0.1908	0.2094

During data preprocessing with the SG algorithm, the Rc of the model decreased and the RMSE increased as the window width increased beyond 5 (**Table 1**), seriously affecting the modeling accuracy.

After comparison of preprocessing results with different window widths, we selected a width of 5 points/time for data preprocessing.

#### 3.2.2 Sample classification

To obtain better modeling results, we used both the Kennard–Stone (KS) algorithm and the joint X-Y distance (SPXY) algorithm to partition samples into the calibration set and the prediction set. The modeling effects obtained after classification with the two algorithms were compared, and the algorithm with a better effect was selected for use in subsequent processing. The division ratio between the calibration and prediction sets was 3:1, which meant that there were 60 samples and 20 samples in the two sets, respectively.

**Table 2** shows the modeling results of power spectrum, absorbance, and transmittance after classification by the KS or SPXY algorithms. These results are not from the final model, so the evaluation indexes associated with the calibration set are used for comparison.

**Table 2:** Modeling results after classification by the KS and SPXY algorithms.

Model	Methods	*R*c	RMSEC
Power	KS	0.8849	0.1601
	SPXY	0.8969	0.1530
Absorbance	KS	0.8404	0.1893
	SPXY	0.8792	0.1702
Transmittance	KS	0.8805	0.1640
	SPXY	0.8908	0.1586

The data in the calibration set obtained with the SPXY algorithm were more correlated and had a lower RMSEC than the data obtained with the KS algorithm (**Table 2**). The calibration set models for power spectrum had higher Rc, lower RMSEC, and higher model quality than the calibration set models for absorbance and transmittance. Subsequent data analysis and processing were based on the SPXY algorithm.

#### 3.2.3 Feature band extraction

The extraction of the feature frequency band is a key aspect of THz time-domain spectral modeling. The full THz band typically contains many variables that have a low correlation with the target value, and there is also collinearity between similar variables. If irrelevant variables are not eliminated and the full frequency band is used for modeling, the model will be complex, and some of the irrelevant variables will lower the modeling accuracy (Liu et al., 2014).

Because too few variables lead to low model accuracy and too many variables result in excessive model complexity, we adopted the stability competitive adaptive re-weighted sampling (SCARS) algorithm to extract feature frequency bands, thereby simplifying the model and improving its efficiency and accuracy. SCARS uses the stability of the variable as a measure, and variables with greater stability are more likely to be selected. Moreover, the frequency band selected remains consistent for each iteration, ensuring that variable selection is stable and fast.

**Figure 6:**
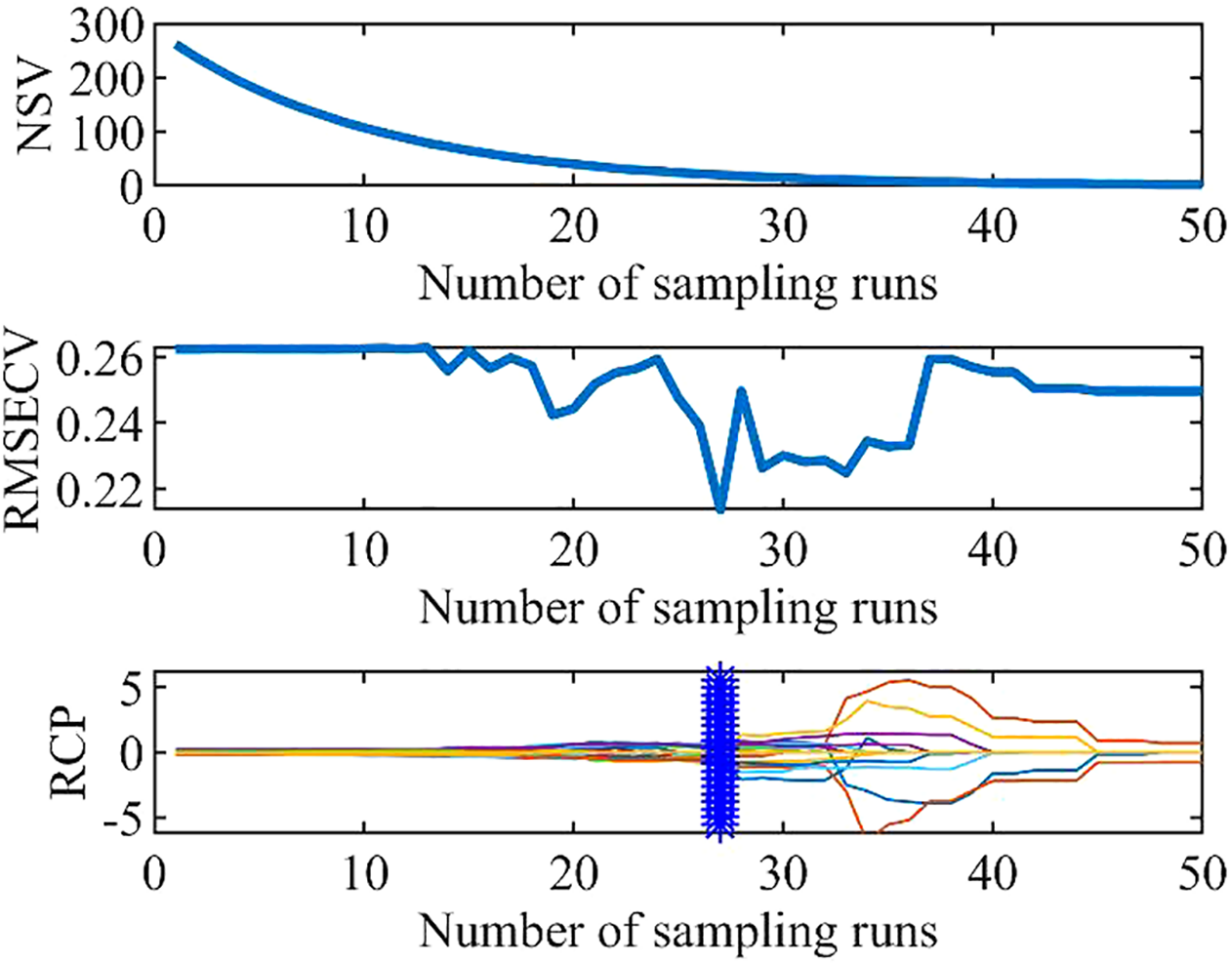
Results of the SCARS algorithm.

The optimal potential band variables are selected by Monte Carlo cross-validation, and the RMSEC is obtained after each cycle. Due to the large number of sampling times, the subset combination with the smallest RMSEC must be selected after several repeated tests for comparison in order to obtain a better combination of characteristic bands. Here, the number of sampling cycles was 50, and the operation results therefore tended to be stable. The results of SCARS are shown in **Figure 6**, using the power spectrum as an example.

The RMSECV of the cross-validation model of the power spectrum reached a minimum of 0.2136 after 27 sampling runs and then gradually increased, indicating that SCARS began to eliminate feature variables that had a large impact on the algorithmic accuracy. Therefore, the subset of feature variables obtained in the 27th run was considered to be optimal, and a total of nine THz feature bands significantly correlated with moisture content were selected.

The absorbance and transmittance feature bands were extracted similarly (**Table 3**).

**Table 3:** esults of feature frequency band extraction.

Model	Times	Select	minRMSECV	Frequency point/THz
Power	27	9	0.2136	0.53, 0.60, 0.76, 0.81, 1.08, 1.14, 1.26, 1.35, 1.45
Absorbance	29	6	0.2081	0.54, 0.59, 1.19, 1.28, 1.34, 1.45
Transmittance	35	7	0.1963	0.51, 0.54, 0.59, 1.24, 1.28, 1.30, 1.46

The extracted feature bands were concentrated around 0.54, 0.59, 1.28, 1.34 and 1.45 THz, which were correlated with peaks or troughs in the THz moisture content curves.

#### 3.2.4 Model analysis

After the extraction of THz feature bands, multiple linear regression models were built by combining the optimized frequency bands of the three optical features with the measured moisture content of the corresponding samples. The modeling results for the single dimensions of power spectrum, absorbance, and transmittance are shown in **Table 4**.

**Table 4:** Results of single-dimension models.

Index	Power	Absorbance	Transmittance
*R*_c_	0.8917	0.9102	0.9039
RMSEC	0.1044	0.1072	0.1061
*R*_p_	0.8996	0.9145	0.8979
RMSEP	0.1482	0.1199	0.1132

The model based on the dimension of absorbance showed the highest correlation between the calibration and prediction sets, reaching 0.9102 and 0.9145, respectively, and the RMSEs of the two sets were 0.1072 and 0.1199, respectively. The model built from the power spectrum dimension had the lowest correlation for the calibration set (0.8917) with an RMSE of 0.1044.

Comprehensive analysis of the single-dimension models showed that the calibration set and the prediction set produced unsatisfactory results: the accuracy and stability were low and could be further improved.

### 3.3 Fusion modeling

#### 3.3.1 Normalization

#### 3.3.2 PCA

After the fusion of spectral features from the three dimensions, the number of feature bands obtained was greatly increased and the data dimension was enhanced, making the model inconvenient. To reduce the model complexity, we used PCA to reduce the data dimensionality after feature fusion.

PCA is a multivariate statistical method that selects a small number of variables from a large number of variables, such that most of the information in the raw data can be replaced by a series of linear transformations (Wang et al., 2017).

The contribution rate and cumulative contribution rate of the principal components after performing data dimension reduction through PCA are shown in **Table 5**.

**Table 5:** Results of PCA algorithm.

Number	Contribution (%)	Cumulative contribution (%)
1	0.3332	0.3332
2	0.2452	0.5784
3	0.1631	0.7415
4	0.1085	0.8500
5	0.0574	0.9074
6	0.0256	0.9330
7	0.0189	0.9519
8	0.0092	0.9611
22	0.0008	1.0000

When the number of principal components was 7, the cumulative contribution reached 0.9519 (>95%), and most of the valid information from the feature fusion was retained. Hence, reducing the raw feature variables from 22 to 7 dimensions can avoid overfitting and reduce model complexity.

#### 3.3.3 Support vector machine (SVM)

SVM based on statistical learning theory has strong learning ability for small samples, high model generalization performance, and the ability to handle high dimensional data, making it particularly suitable for dealing with small samples, nonlinearity, and high dimensional problems encountered in practical applications. Therefore, the dimensionality-reduced fused feature variables were used for regression modeling with SVM.

The regression model was built using the LibSVM package in Matlab and was developed and designed by Professor Lin Chih-Jen of National Taiwan University. This model is simple and easy to use, and it can solve problems of classification, regression, and distribution estimation. Among the many nuclear functions, the radial basis nuclear function (RBF) has low computational complexity and is a reasonable first choice (Wang et al, 2022).

The main parameters of an RBF model are the nuclear function of the Gamma function parameter *g*, the error penalty factor *C*, and the loss function *p*. Parameter settings can be adjusted based on the output results and the law of curve changes until prediction accuracy meets the requirements.

**Figure 7:**
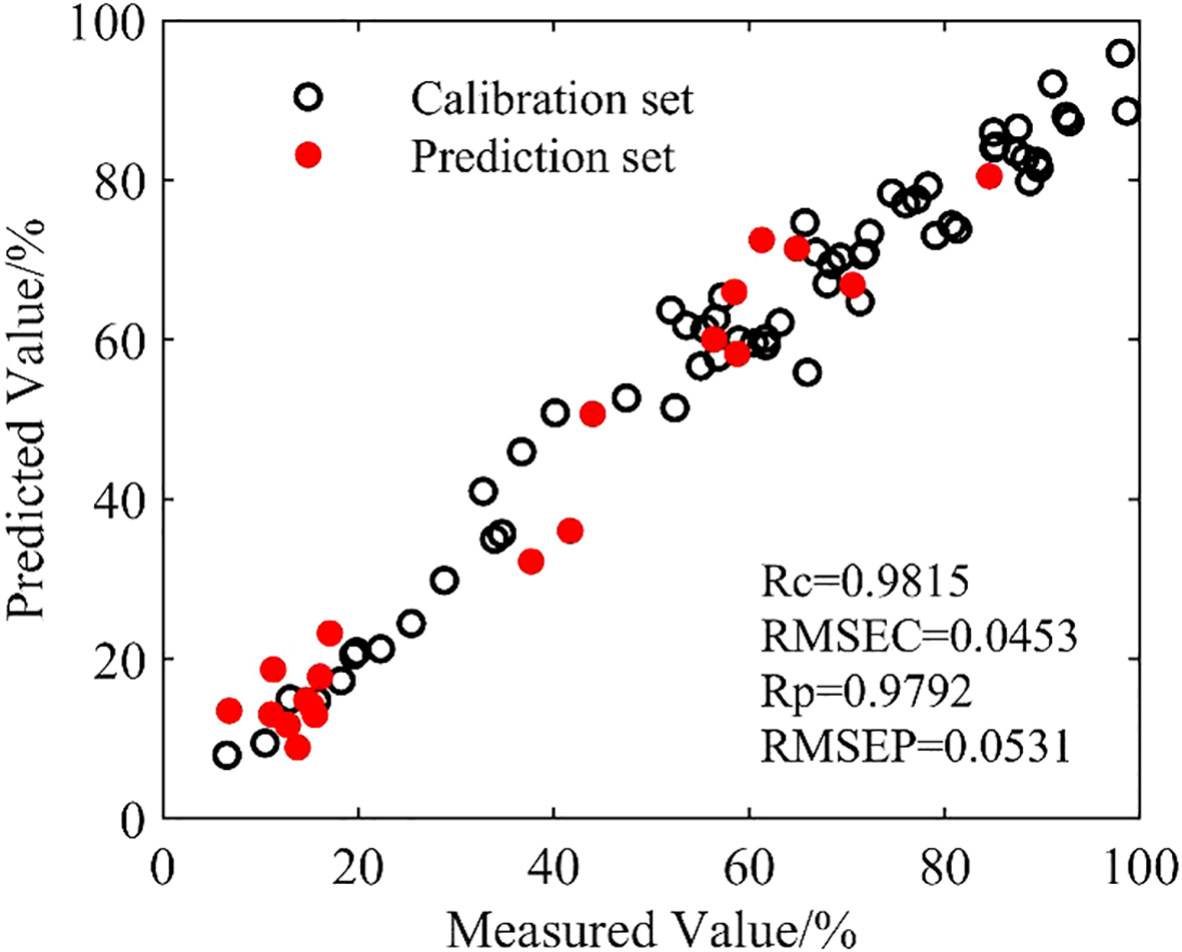
Scatter plot of SVM.

A cross-validation approach was used to select the best parameters, and the highest Rc was 0.9815 when *g*, *C* and *p* were 8.65, 2.41 and 0.01, respectively. The results of fusion modeling are shown in **Figure 7**.

The scatterplot relationship analysis showed that the Rc and Rp were 0.9815 and 0.9792, respectively, higher than the highest values of 0.9102 and 0.9145 from the single-dimension models. The RMSRC and RMSRP were 0.0453 and 0.0531, respectively, lower than those of all the single-dimension models. Hence, the results of the fusion model created from different dimensions by SVM outperformed the results of all the single-dimensional models.

## 4 Conclusions

The relationships among the THz spectra in the power spectrum, absorbance, and transmittance were studied and modeled separately. Finally, a fusion model for tomato leaf moisture content prediction was developed by fusing the feature bands from the three dimensions using SVM. In the frequency range of 0.5–1.5 THz, leaf moisture content level was positively correlated with absorbance and negatively correlated with both transmittance and frequency. At the same frequency, as the water level decreased, the power spectrum and absorbance decreased with significant negative correlations, and transmittance increased with a significant positive correlation. The model based on absorbance feature frequency produced the best results, with a correlation coefficient of 0.9145 and a root mean squared error of 0.1199 for the prediction set.(1) The prediction set correlation coefficient of the fusion model was 0.9792, an improvement in accuracy of 7.1% compared with the absorbance model, and its root mean square error was 0.0531, indicating a better prediction effect.

## Data availability statement

The original contributions presented in the study are included in the article/supplementary material. Further inquiries can be directed to the corresponding author.

## Author contributions

Conceptualization, YZ, LH, and XW; methodology, YZ, PW, XW, and YW; formal analysis, PW, YZ and YW data curation, PW and YZ; writing—original draft preparation, YZ, YW, and PW; writing—review and editing, YZ and YW. All authors contributed to the article and approved the submitted version.
